# Learning of veterinary professionals in communities: a thesis report

**DOI:** 10.1007/s40037-013-0073-0

**Published:** 2013-08-09

**Authors:** Esther de Groot

**Affiliations:** 1 Chair Quality of Veterinary Medical Education, Faculty of Veterinary Medicine, Universiteit Utrecht, Yalelaan 1, 3584 CL Utrecht, the Netherlands; 2 Present Address: Steve Bikostraat 298, 3573 BH Utrecht, the Netherlands

**Keywords:** Evidence based practice, Learning communities, Critically reflective work behaviour

## Abstract

Veterinary professionals can improve on how they continue learning through critically reflective work behaviour in communities. In this way participation in communities might support the transition to evidence-based practice.

## Introduction

Veterinary professionals are required to continue learning, and need to increasingly practice in an evidence-based manner. We investigate how continued learning through critically reflective work behaviour (CRWB) takes place in communities, and explore how participation in learning communities might at the same time support the transition to evidence-based practice. CRWB consists of several aspects that help learning to occur: openness about mistakes, challenging groupthink, asking for feedback, experimentation and critical opinion sharing.

## Methods

For all veterinarians in the Netherlands, their CRWB and research utilization were measured with a questionnaire, and the response was analyzed with Structural Equation Modelling. Next, we analyzed dialogues for the CRWB aspects with a self-developed framework in seven different communities. Finally, we explored what factors are relevant to entice professionals into CRWB with a Delphi study.

## Results

1. The intended, idealized, purpose of learning communities seems not to be met yet. Veterinary professionals in communities infrequently address each other’s substantiated arguments and reflections on clinical policies, missing benefits from learning in social interaction;
2. In addition to aspects of CRWB that were recognized in earlier studies, research utilization should be considered a new aspect of CRWB;
3. Giving access to full-text versions of research papers and a short training in searching the literature did not lead to changes in practitioners dialogues;
4. Thirteen factors were found on how professionals can be enticed into CRWB; for example: ‘diversity in the group, especially in expertise, to achieve different points of view’.

## Discussion

If learning communities of veterinary professionals are to fulfil their potential, it is essential to address each other’s substantiated arguments and reflections on clinical policies more often. Adjustments in the design of their meetings are needed to ensure that participating in communities will help to make the transfer to evidence-based practice. Attention should also be paid to personal attributes such as a perceived need for lifelong learning and personal epistemologies.

## Conclusion

To make the transfer to evidence-based practice it is essential to have critically reflective discussions. For this, moderators are important. To prepare future professionals, a change in pedagogical practices is necessary. In addition to training in literature searching and appraisal skills, there is a need for small-group learning with a focus on clinical uncertainty.

